# The Galaninergic System: A Target for Cancer Treatment

**DOI:** 10.3390/cancers14153755

**Published:** 2022-08-01

**Authors:** Manuel Lisardo Sánchez, Rafael Coveñas

**Affiliations:** 1 Laboratorio de Neuroanatomía de los Sistema Peptidérgicos (Lab. 14), Instituto de Neurociencias de Castilla y León (INCYL), Universidad de Salamanca, c/Pintor Fernando Gallego 1, 37007 Salamanca, Spain; covenas@usal.es; 2 Grupo GIR USAL: BMD (Bases Moleculares del Desarrollo), University of Salamanca, 37007 Salamanca, Spain

**Keywords:** galanin, galanin receptor, galanin receptor antagonist, galanin receptor agonist, neuroendocrine tumors, signaling pathways

## Abstract

**Simple Summary:**

Peptidergic systems play an important role in cancer progression. The galaninergic system (the peptide galanin and its receptors: galanin 1, 2 and 3) is involved in tumorigenesis, the invasion and migration of tumor cells and angiogenesis and it has been correlated with tumor stage/subtypes, metastasis and recurrence rate in many types of cancer. Galanin exerts a dual action in tumor cells: a proliferative or an antiproliferative effect depending on the galanin receptor involved in these mechanisms. Galanin receptors could be used in certain tumors as therapeutic targets and diagnostic markers for treatment, prognosis and surgical outcome. This review shows the importance of the galaninergic system in the development of tumors and suggests future promising clinical antitumor applications using galanin agonists or antagonists.

**Abstract:**

The aim of this review is to show the involvement of the galaninergic system in neuroendocrine (phaeochromocytomas, insulinomas, neuroblastic tumors, pituitary tumors, small-cell lung cancer) and non-neuroendocrine (gastric cancer, colorectal cancer, head and neck squamous cell carcinoma, glioma) tumors. The galaninergic system is involved in tumorigenesis, invasion/migration of tumor cells and angiogenesis, and this system has been correlated with tumor size/stage/subtypes, metastasis and recurrence rate. In the galaninergic system, epigenetic mechanisms have been related with carcinogenesis and recurrence rate. Galanin (GAL) exerts both proliferative and antiproliferative actions in tumor cells. GAL receptors (GALRs) mediate different signal transduction pathways and actions, depending on the particular G protein involved and the tumor cell type. In general, the activation of GAL_1_R promoted an antiproliferative effect, whereas the activation of GAL_2_R induced antiproliferative or proliferative actions. GALRs could be used in certain tumors as therapeutic targets and diagnostic markers for treatment, prognosis and surgical outcome. The current data show the importance of the galaninergic system in the development of certain tumors and suggest future potential clinical antitumor applications using GAL agonists or antagonists.

## 1. Introduction

**Figure 1:**
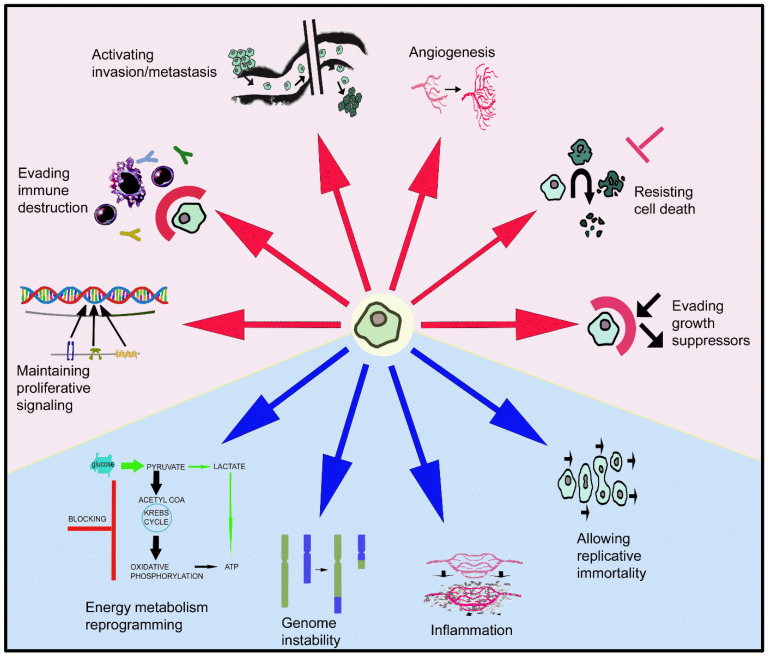
Ten keys of cellular/tissue behavior that make a cell a cancer cell, contrary to its normal biological destiny, leading to the formation of a primary tumor and later a secondary one. Red arrows show the involvement of the galaninergic system in these mechanisms: note that GAL is involved in six of them.

The GLOBOCAN 2020 database (World Health Organization (WHO)) states that of the 7,794,798,844 inhabitants of our planet, 19,292,789 of them were diagnosed with some type of cancer and 9,958,133 died, with prevalence cases at 5 years of 50,550,287. Female breast cancer is the most diagnosed cancer and the leading cause of cancer death is lung cancer (1.8 million deaths) [1]. In 2040, 28.4 million patients suffering from cancer are expected in the world [1]. These data are sufficiently representative of the health problem that cancer represents today. Cells, escaping from normal behavior, acquire distinctive characters (evading growth suppressors, maintaining proliferative signaling, allowing replicative immortality, resisting cell death, activating invasion/metastasis, inducing angiogenesis) that make them cancerous [2] (Figure 1). Moreover, the reprogramming of energy metabolism and evasion of immune destruction have also been added to the previous hallmarks of cancer [2]. These behaviors arise from the instability of the genome that produces genetic diversity, and inflammatory mechanisms that promote the multiple actions described above (Figure 1). Tumors are not currently considered as simple masses of cancer cells; they are more complex in that they contain a repertoire of apparently normal recruited cells that contribute to the acquisition of distinctive features by regulating the tumor microenvironment [2]. The full knowledge of the previously mentioned hallmarks will help to develop new therapeutic strategies against cancer.

Neuropeptides such as galanin (GAL), angiotensin II, apelin, adrenomedullin, endothelin-1, bombesin, orexin, substance P, neuropeptide Y, calcitonin gene-related peptide, vasoactive intestinal peptide and neurotensin are involved in cancer [3,4,5,6,7,8,9]. The overexpression of the peptidergic systems has been involved in the progression of some types of cancer [3,5,9]. In general, the mentioned peptides promote the proliferation, invasion and migration of tumor cells, angiogenesis and lymphangiogenesis and exert an antiapoptotic effect in these cells. However, other peptides exert an anticancer action; this is the case of the heptapeptide angiotensin (1–7) which blocks cell proliferation and angiogenesis. For these reasons, it is necessary to investigate the roles played by the peptidergic systems in cancer in more depth. This line of research has been developed over the last several years and the knowledge of the roles played by peptides in tumor progression has notably increased [9]. It is important to note that the galaninergic system has been involved in six (e.g., proliferative action, invasion, metastasis, angiogenesis) of the ten cancer hallmarks previously mentioned (Figure 1). Unlike what happens with other peptides (e.g., substance P, neurotensin), which exclusively exert a proliferative action on tumor cells [9,10], GAL exerts this proliferative action, but also suppresses the development of certain types of cancer (e.g., neuroblastoma, head and neck squamous cell carcinoma, gastric cancer) [11,12,13]. Thus, due to the crucial role that GAL plays in cancer, the aim of this review is to show the involvement of the galaninergic system in this disease and to suggest potential therapeutic strategies to block the development of tumors using GAL receptor antagonists or agonists. The latter is an important point that must be developed in the future to identify potential antitumor targets and to better evaluate the involvement of GAL in cancer.

## 2. The Galaninergic System: Galanin and Its Receptors

**Figure 2:**
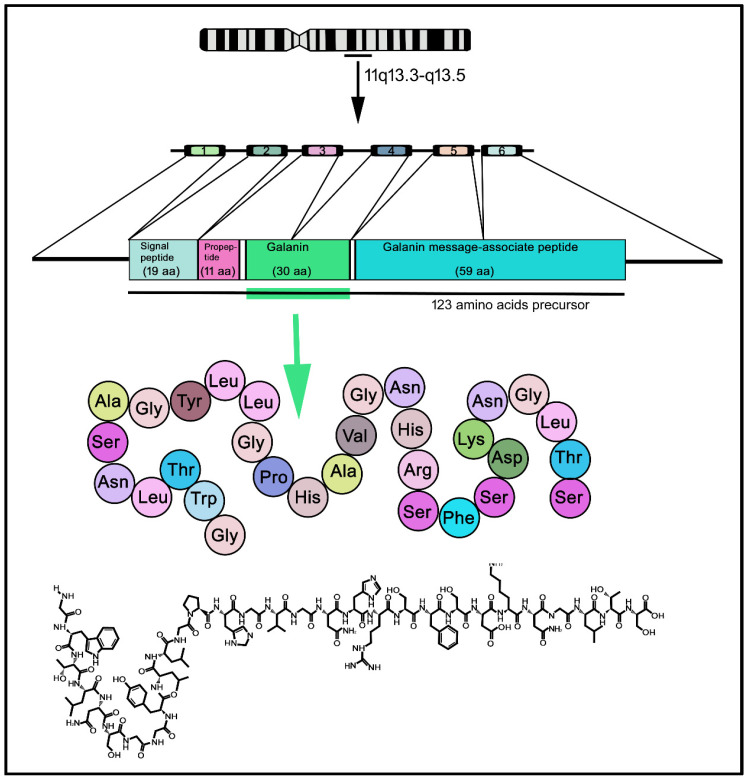
Transcription–maturation–translation processing of GAL, from human chromosome 11. Human GAL contains 30 amino acids residues. 1–6: exons; aa: amino acids.

GAL was discovered in porcine intestinal extracts and contains 29 amino acids [14]; however, in humans, the peptide contains 30 amino acid residues (Figure 2) and, unlike porcine GAL, the carboxy-terminus is not amidated [15,16,17]. The amino acid sequence of GAL is highly conserved among species (almost 90%) [18]. The C-terminus of GAL is involved in its receptor-binding affinity and the N-terminus is crucial for its biological activity [19]; the fifteen N-terminal residues of GAL are highly conserved throughout evolution [20]. GAL and other peptides (GAL message-associated peptide (GMAP), GAL-like peptide (GALP), alarin) belong to the GAL family of peptides. In addition, the peptide spexin (neuropeptide Q, 14 amino acids) is the most recently discovered member of this family; spexin has been shown to be involved in reproduction, nociception, renal function and energy homeostasis [21]. GALP, an endogenous ligand that activates the three known types of GALRs, was isolated from the porcine hypothalamus, contains 60 amino acids and is involved in reproduction and energy homeostasis [22,23]. Alarin (25 amino acids) is a splice variant of GALP mRNA [24]. The human chromosome 11q13.3-q13.5 contains the pre-pro-GAL gene-encoding GAL, which shows five introns and six exons, which in turn are translated into a pre-prohormone (123 amino precursor) containing the signal peptide, GAMP and GAL [17,25] (Figure 2). Some oncogenes have been located in the abovementioned region, which is also the breakpoint for the translocation t (11; 14) (q13; q32) in diffuse B-cell lymphoma and chronic lymphocytic leukemia [26]. The gene spans 6.5 kb and its first exon only encodes the 5′ untranslated sequence. In the pre-pro-GAL gene, its 5-prime flanking sequence shows a TATA box preceded by binding sites for transcription factors (e.g., NF-κB) and contains a CT-rich region that is flanked by two Alu repeats-, 2.3 kb upstream of the transcriptional start site; the region (500 bp) preceding this site contains 79% CG [27]. GALP and alarin are encoded by the pre-pro-GALP gene, which is located on the human chromosome 19q13.43 and comprises six exons [28]. The region encoding GALP is contained in exons 2–5 and alarin is formed when post-transcriptional splicing leads to the exclusion of exon 3, resulting in a frame shift and a novel precursor peptide [24].

**Figure 3:**
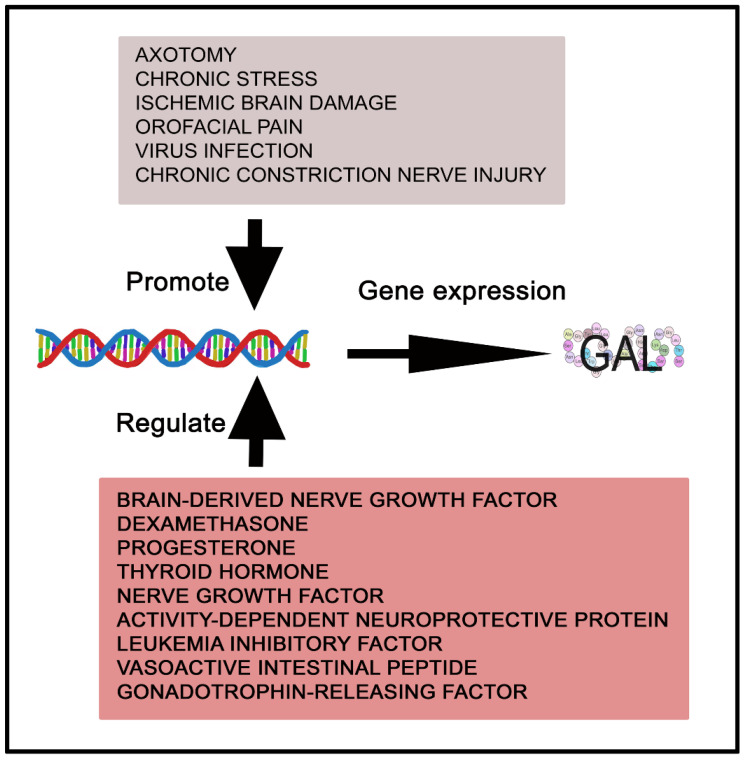
Pathological situations and bioactive molecules promoting and regulating, respectively, the expression of GAL.

The galaninergic system (GAL and GAL receptors (GALRs)) is widely distributed by the mammalian gastrointestinal tract, testis, ovary, uterus, kidney and heart, and by the immune, endocrine, peripheral and central nervous systems (e.g., endocrine pancreas, pituitary gland, paravertebral sympathetic ganglia, myenteric plexus, glial cells, dorsal root ganglion, spinal cord, brainstem, thalamus, hypothalamus, hippocampus, amygdala) [25,29,30,31,32,33,34,35,36]. The half-life of GAL in plasma is about five minutes and GAL coexists with many other neuroactive substances (e.g., enkephalin, vasopressin, calcitonin gene-related peptide, substance P, neuropeptide Y, cholecystokinin, growth hormone, luteinizing hormone-releasing hormone, dopamine, glutamate, noradrenalin, serotonin, acetylcholine) [29,37,38,39,40,41,42,43,44]. In general, GMAP in the rat central nervous system showed a similar profile of expression to GAL; however, GALP and alarin showed a more restricted expression than GAL [45]. Due to the widespread distribution of the galaninergic system by the whole body, GAL has been involved in many physiological actions after binding to specific G protein-coupled receptors: smooth muscle contraction, acetylcholine release inhibition, energy metabolism, food and water intake, hyperglycemia, osmotic and metabolic homeostasis, spinal reflexes, injury response, nociception, reproduction, memory, cognition, learning, arousal, sleep, neural growth, glucose-induced insulin release inhibition and respiratory, cardiovascular, neuroendocrine and gastrointestinal mechanisms [8,14,18,20,25,29,33,38,46,47,48,49,50]. Moreover, GAL regulates the level of growth hormone, prolactin, dopamine, pancreatic peptide, luteinizing hormone, luteinizing hormone-releasing hormone, somatostatin and insulin [18,42,51,52,53]. GAL acts as a neurotransmitter and neuromodulator in the central nervous system and the peptide has been involved in several diseases (e.g., anxiety, depression, stroke, alcoholism, Alzheimer’s disease, Parkinson’s disease, epilepsy); the galaninergic system also plays an important role in inflammatory bowel diseases and diabetes [18,20,25,54,55,56,57,58]. In addition to the nervous system actions mediated by the galaninergic system (e.g., GAL exerts a neuroprotective action in the hippocampus and favors neurite outgrowth) [49,50], GAL also mediates non-neural functions including the emerging roles played by the peptide in tumorigenesis [20] and in tumor-infiltrating immune cells (e.g., glioma-associated macrophages, microglia, neutrophils) [30]. GAL regulates the expression of chemokines (CCL2, CCL3, CCL5, CXCL8) and anti-inflammatory cytokines (tumor growth factor-β, interleukin-10, interleukin-1Ra) in macrophages [59]. The expression of GALRs in tumor-related immune cells suggests that GAL regulates the homeostasis of the tumor microenvironment. In humans, the expression of GAL is regulated in a cell type-specific manner by the brain-derived nerve growth factor, dexamethasone, progesterone, thyroid hormone, nerve growth factor, activity-dependent neuroprotective protein, leukemia inhibitory factor, vasoactive intestinal peptide and gonadotropin-releasing factor [20]. Protein kinase A (PKA) and protein kinase C (PKC) are inducers of the expression of the GAL gene, and the expression and release of GAL is promoted by axotomy, chronic stress, ischemic brain damage, orofacial pain, virus infection and chronic constriction nerve injury [20] (Figure 3).

GALRs (GAL 1 receptor (GAL_1_R), GAL 2 receptor (GAL_2_R), GAL 3 receptor (GAL_3_R)) belong to the rhodopsin-like (class A) G protein-couple receptor family (seven transmembrane receptors or 7TM) [60]. They contain three extracellular loops, three intracellular loops, an extracellular N-terminus and three intercellular loops [60,61]. The helix 8 acts as a conformational switch at the C-terminus [62]. GALRs have sequence homologies in the transmembrane region: GAL_1_R-GAL_3_R (33%) and GAL_2_R-GAL_3_R (54%) [20], whereas human GAL_3_R and GAL_2_R respectively show 89% and 92% sequence homology with their receptor homologs present in the rat [63]. Human GAL has tens of nanomolar affinity at GAL_3_R, subnanomolar to nanomolar affinity at GAL_2_R and subnanomolar affinity at GAL_1_R [64]. Although the structure of GALRs is quite similar, different binding characteristics and intracellular signaling pathways have been reported after the activation of these receptors by ligands [60,61]. Thus, the lengths of the N-terminus (which plays an important role in the binding of ligands) and C-terminus are different in GALRs (C-terminus: GAL_1_R, 37 residues; GAL_2_R, 30; GAL_3_R, 13; N-terminus: GAL_1_R, 47 residues; GAL_2_R, 80; GAL_3_R, 62) [60]. The physiological actions of GAL are mediated by GAL_1_R, GAL_2_R and GAL_3_R; several signaling pathways are activated after the binding of GAL to these receptors: the stimulation of phospholipase C (PLC, mediated by GAL_2_R) or the inhibition of cyclic adenosine monophosphate (cAMP)/PKA (mediated by GAL_1_R/GAL_3_R) [26]. Moreover, GAL_2_R mediates the inhibition of adenylate cyclase (AC) via coupling to Gi type G protein [65,66]. GALR type is determined by the region between the transmembrane helix 7 and the extracellular loop 2 (a variable region affecting the binding of ligands) and by the cavity size (e.g., the GAL_3_R binding cavity is narrower than that observed in GAL_1_R or GAL_2_R) [60]. Human GAL_2_R and GAL_3_R genes have respectively been localized in chromosomes 17q25 and 22q12.2-13.1 [66].

GAL_1_R was isolated from a human melanoma cell line [67]. It is coupled to Gβγ/Gαi signaling pathways and promotes, via a PKC-independent mechanism, the activation of mitogen-activated protein kinases (MAPKs) [17,68]. Moreover, the activation of GAL_1_R inhibited AC activity via an interaction with G-proteins (Gαi/αo), leading to G protein-coupled inwardly-rectifying potassium (GIRK) channels opening [32,67,69]. GAL_1_R activation can also inhibit the transcription factor cAMP regulatory element binding protein (CREB)-dependent signaling pathway [70], and the expression of GAL_1_R (but not that of GAL_2_R or GAL_3_R) was controlled by cAMP via CREB [71,72]. The GAL_1_R gene (located in chromosome 18q23) in humans shows three exons that are translated into a long protein containing 349 amino acids; GAL_1_R homology is high between species (e.g., in mouse, 93% of the residues are identical to those observed in humans) [73]. GAL_1_R has been located in the central (e.g., cortex, amygdala, hippocampus, thalamus, hypothalamus, locus coeruleus, medulla oblongata, spinal cord) and peripheral (e.g., dorsal root ganglion) nervous systems [33,34] and in the gastrointestinal tract [67,74]. 

GAL_2_R was first identified in the rat central nervous system [35,75,76] and was cloned in rat hypothalamic cells for the first time [35]. GAL_2_R contains His252/His253 (transmembrane domain 6) and Phe264/Tyr271 (extracellular loop 3) residues, which play a crucial role in the binding of ligands and in the activation of the receptor [77]. The sequence of human GAL_2_R shows a high homology with that observed in the rat (85–92%) and it was 39% identical to human GAL_1_R [33,63,78]. In the rat, GAL_2_R shows 38% amino acid identity with GAL_1_R [35]. In comparison with GAL_1_R, the distribution of GAL_2_R is more widespread since it has been observed in the nervous system (piriform cortex, dentate gyrus, amygdala, hypothalamus, mammillary nuclei, spinal cord), skeletal muscle, liver, testis, ovary, uterus, spleen, heart, kidney, lung, gastrointestinal tract and pituitary gland [33,35,63,79,80]. GAL_2_R mRNA expression has been reported in the neocortex, dentate gyrus, hypothalamus, cerebellar cortex, substantia nigra, vestibular complex and dorsal root ganglion [7,80,81]. GAL_2_R expression was modified in the thalamus and cerebral cortex during brain development; this suggests that the receptor is involved in important mechanisms during the establishment/maturation of synaptic circuits and during neural damage/repair in the mature nervous system [82]. GAL_2_R activates the G protein (Gαq/11) pathway by triggering the intracellular phosphoinositol turnover, the activity of PLC and the release of Ca^2+^ into the cytoplasm [35,63,68]. GAL_2_R, via PKC and G protein (Gαo), activated MAPKs, favoring the downstream phosphatidylinositol 3-kinase (PI3K)-dependent phosphorylation of PKB and blocked the activity of caspases 3 and 9 [68,83]. GAL, via GAL_2_R, induced the nuclear factor of activated T-cells and the cytoplasmic 2 (NFATC2)-mediated transcription of cyclooxygenase 2 and GAL, leading to the secretion of prostaglandin E2 and GAL, which favored cell invasion and neuritogenesis, respectively [84]. GAL_2_R can block forskolin-stimulated cAMP production; this suggests the activation of Gαi/αo [66,85], and CREB [70]. GAL, via GAL_2_R, activated extracellular-regulated protein kinase (ERK) and the phosphorylation of the serine/threonine kinase Akt signaling pathway [86]. The activation of GAL_2_R promoted, via the Akt (PKB) pathway, cell survival and proliferation; both processes were MAPK1/MAPK3-dependent [87]. GAL_2_R mediated the neuroprotective effect promoted by GAL after injury and also activated PKC, PLC and ERK via Gq/11 [17,68,88]; this means that after binding to GAL_2_R, GAL agonists could be used to treat neurodegenerative diseases (e.g., multiple sclerosis) [49]. This is an important line of research that must be developed in the future; in particular, research must be focused on the search of GAL_2_R-specific agonists.

GAL_3_R was first isolated from rat hypothalamic cDNA libraries [89]. Human GAL_3_R (368 amino acids long) shows 36% amino acids identity with human GAL_1_R, 58% with human GAL_2_R and 90% with rat GAL_3_R [63]. The distribution of GAL_3_R (olfactory cortex, hippocampus, hypothalamus, medulla oblongata) is more restricted than that reported in the brain for GAL_1_R or GAL_2_R [33,63,77,89,90,91]. GAL_3_R mRNA has been located in the amygdala, periaqueductal gray, locus coeruleus, brainstem reticular formation, spinal cord, pancreas, adrenal gland and testis [63,91]. GAL_3_R promotes the activation of Gαi/αo, blocking AC activity and opening GIRK channels [63,90]. Spexin binds to human GAL_2_/_3_Rs (not to GAL_1_R), exerting a higher potency toward GAL_3_R than GAL [21,92].

GAL agonists or antagonists (e.g., galantide, M35, M40, C7) have been used for the treatment of several disorders: GAL antagonists have been administered for the treatment of food intake disorders and Alzheimer’s disease, whereas GAL agonists have been used for the treatment of chronic pain [18,93]. Some fragments of GAL (GAL1-15; GAL1-16, GAL1-29), exerting physiological actions through GALRs (e.g., mood or cardiovascular regulation, alcohol intake), have been reported [94,95,96,97]. The conformational changes observed in GAL_1_R lead to a higher affinity of this receptor for GAL1-15 than for GAL, increasing the signaling (mediated by Gi/o) and decreasing AC activity and CREB level [98]. GALRs may form heteromers with each other and with other types of G protein-coupled receptors in the central nervous system [99]. Thus, the GAL_1_R/GAL_2_R heteroreceptor complex [98] and heteromers of GALRs with alpha2-adrenoceptors and 5-hydroxytryptamine (HT), dopamine 1, neuropeptide Y1 or Y2 receptors have been reported [20]. The formation of the heterotrimer GAL_1_R-GAL_2_R-5-HT1A receptor complex could explain why GAL1-15, but not GAL1-29, antagonistically moderated the serotonin receptor [99]. In addition, this heterotrimer has been suggested as a potential target to reverse the actions mediated by fluoxetine on memory mechanisms [94,100]. Thus, heteromers can alter the recognition of GAL ligands, and they are promising new targets for therapeutic interventions.

## 3. The Galaninergic System and Cancer

Peptides and their receptors are one of the molecular bases for the therapeutic targeting of tumors [101]. The galaninergic system is expressed in normal tissues and, in cancer cells, is involved in tumorigenesis, invasion and migration (metastasis) [30,36,39,101,102,103,104,105,106,107,108,109,110,111,112], although in some tumors, GAL and GALRs are silenced [113]. This system has been observed in neuroendocrine (e.g., phaeochromocytoma, pituitary adenoma, gangliocytoma, paraganglioma, neuroblastoma) and non-neuroendocrine (e.g., glioblastoma and other brain tumors, melanoma, basal cell carcinoma, head and neck squamous cell carcinoma, embryonic carcinoma, colon cancer, breast cancer, gastrointestinal cancer, prostate cancer) tumors [30,36,39,75,101,102,103,104,105,106,107,108,109,110,111,112,114,115,116,117,118,119,120,121]. For example, in squamous cell carcinoma, GAL_1_R was involved in tumor suppression and GAL_2_R favored tumor development and decreased survival [122,123]. GAL exerted a tumor-reducing effect in experimental murine models (gastrointestinal cancer), but in other models (adenoma formation), GAL promoted cell proliferation and tumor formation [101]. Thus, GAL can promote or inhibit the development of tumors; this is an important characteristic of the galaninergic system: to exert both proliferative and antiproliferative actions on tumor cells. Importantly, GAL/GALR expression has been correlated with tumor subtypes (colon carcinoma, squamous cell carcinoma, neuroblastic tumors, pituitary adenoma) or with tumor stage [101] and the activation of GAL_1_R was generally antiproliferative, whereas the activation of GAL_2_R showed antiproliferative or proliferative effects [101]. The stage and tumor size in colon cancer have been related to the GAL mRNA level: the higher the GAL expression, the shorter the disease-free survival [30,106]. In general, the data reported above suggest that the galaninergic system is a promising target for the diagnosis, prognosis and treatment of tumors expressing GAL and GALRs. In this section, the involvement of this system in neuroendocrine tumors (phaeochromocytomas, insulinomas, neuroblastic tumors, pituitary tumors, small-cell lung cancer), gastric cancer, colorectal cancer, head and neck squamous cell carcinoma and glioma will be reviewed as well as other cancer types in which the galaninergic system has been less studied.

### 3.1. Galanin and Neuroendocrine Tumors 

Neuroendocrine tumors (NETs) are a very heterogeneous tumor group including: (1) carcinoid gastroenteropancreatic tumors; (2) non-carcinoid gastroenteropancreatic tumors (vasoactive intestinal peptide (VIP)oma, gastrinoma, insulinoma); (3) catecholamine-secreting tumors (neuroblastoma, sympathoblastoma, ganglioneuroblastoma, ganglioneuroma, paraganglioma, phaeochromocytoma); (4) chromophobe pituitary tumors; (5) medullary carcinoma of the thyroid; (6) Merkel cell tumors; and (7) small-cell lung cancer. NETs originate from neuroendocrine cells, which release peptides (e.g., GAL, somatostatin, pancreatic polypeptide, chromogranins) and express their corresponding receptors [124,125,126]. Thus, a high expression of peptidergic receptors has been reported in NETs for neurotensin, gastrin-releasing peptide, cholecystokinin, somatostatin and vasoactive intestinal peptide [125]. Importantly, the expression of the peptidergic systems in NETs has been correlated with prognosis and tumor stage [127].

Regarding the galaninergic system, many data demonstrated its involvement in NETs pathophysiology and carcinogenesis; for example, high doses of estrogens or dopamine agonists reversed rat pituitary hyperplasia and decreased the expression of GAL, suggesting that the peptide acted as a proliferative agent [128,129,130,131,132]. GAL expression is restricted to some NETs [107]: the peptide was observed in adrenal phaeochromocytoma (62%), jugulo tympanic paraganglioma (40%) and carotid body paraganglioma (18%), but it was not found in metastatic or recurrent paraganglioma, extra-adrenal phaeochromocytoma and carcinoid tumor [107,108]. Moreover, endocrine tumors from gastrointestinal tract, pancreas and lung did not show GAL [107]. This means that the utility of GAL as a diagnostic marker is limited to certain NETs. In this section, the involvement of the galaninergic system in those NETs (phaeochromocytoma, insulinoma, neuroblastic tumor, pituitary tumor, small-cell lung cancer) expressing this system will be reviewed (Table 1). The methodology (e.g., immunohistochemistry, in situ hybridization, Western blot) applied in the studies appearing along the text in different tables is reported. However, it is important to note that antisera directed against G protein-coupled receptors (including GALRs) are frequently unspecific [133,134]; accordingly, the findings found regarding GALRs should be taken with caution and only accepted when using valid controls, with the specificity of these antisera fully confirmed. 

#### 3.1.1. Phaeochromocytoma

GAL and GALRs have been observed in human phaeochromocytomas (Table 1). Compared with normal adrenal glands, the concentration of GAL was much higher in phaeochromocytomas; however, the authors of the study reported that in both phaeochromocytoma patients and normal individuals, the concentration of GAL in plasma was below the detection limit of the assay (less than 10 pmol/liter) [144]. The last observation is surprising, since GAL plasma levels are usually not below the detection limits of the assays. In the latter study, GAL was localized in 5 of 11 of the phaeochromocytomas studied, and in normal adrenal glands, the peptide was only observed in a few cortical nerve fibers/chromaffin cells. A high GAL_2_R mRNA expression was observed in human phaeochromocytomas [143] and GAL inhibited the proliferation of phaeochromocytoma tumor cells [141]. GAL blocked the proliferation of rat PC 12 cells in which the expression of mRNAs encoding the three GALRs, but not GAL mRNA, was observed [141].

#### 3.1.2. Insulinoma

Insulinomas appear sporadically or can be related with multiple endocrine neoplasia type 1 (MEN1 syndrome: an autosomal dominant condition due to MEN1 gene inactivating mutations) [161]. This syndrome is characterized by the presence of tumors in duodenum/endocrine pancreas, anterior pituitary adenomas and primary hyperparathyroidism, with gastrinomas and insulinomas being the most common functioning islet cell tumors [161]. The expressions of GALRs and GAL_1_R have respectively been reported in RINm5F [162] and Rin14B [32] insulinoma cells. GAL (released from sympathetic nerve terminals located in the endocrine pancreas) in insulinoma beta TC-1cells (mouse) blocked the expression of the pro-insulin gene promoted by glucagon-like peptide-I (7-37) [139] (Table 1). It has been reported that GAL did not block the secretion of insulin by simply decreasing the Ca^2+^ level [163]. In the RINm5F insulinoma cell line, GAL inhibited the activity of AC and moderately suppressed the accumulation of insulin, but did not affect cell proliferation [138]; Gi3, a G protein coupled to GALRs, was involved in this inhibition [53]. In pancreatic beta-cells, GAL blocked the secretion of insulin and the activity of AC via pertussis-toxin-sensitive G proteins [53]. Finally, the chimeric peptide M35 (galanin (1-13)-bradykinin (2-9) amide) showed a dual effect depending on the concentration administered: acting as a GALR antagonist (at low concentrations) or as a GALR agonist (at high concentrations) [93].

#### 3.1.3. Neuroblastic Tumor

The expression of GAL mRNA, GAL immunoreactivity and GAL binding sites has been reported in neuroblastic tumors [136,137] (Table 1). Neuroblastoma and ganglioneuroma are neuroblastic tumors, and in both, no correlation between prognosis or tumor markers and the concentration of GAL has been reported [136]. However, a low level of GAL binding sites has been correlated with survival [136] and GAL/GALR expression has been related to the tumor differentiation stage [137].

Neuroblastoma is the result of an aberrant sympathetic nervous system development, usually arising from the paraspinal ganglia or adrenal medulla [116,164]. Thus, neuroblastoma appears in very young children (median age: 17 months; 10.2 cases/million children under 15 years) [165,166]; GAL and GAL mRNA have been detected in this disease [12,104]. The coexistence of GAL and beta-amyloid peptide in dense core secretory vesicles has been reported in the human neuroblastoma IMR32 cell line; this finding suggests that both substances are involved in the regulation of brain functions [140]. Moreover, GAL_1_R and GAL_3_R are highly expressed (immunoreactivity) in neuroblastoma, whereas the presence of GAL_2_R mRNA is less common than that of GAL_1_R mRNA [104,137]. By contrast, the immunoreactivity for both GAL_1_R and GAL_3_R decreased in ganglioneuromas [137]. GAL, GAL_2_R and GAL_3_R mRNAs were detected in the rat neuroblastoma B104 cell line, but not GAL_1_R mRNA [141]. It is important to note that the galaninergic system (by autocrine/paracrine mechanisms) exerts an anticancer action or a proliferative effect on neuroblastoma tumor cells; these effects are mediated by different GALRs, which induce different signaling pathways after the binding of GAL [141]. Thus, GAL promoted the growth and development of human neuroblastoma in an autocrine/paracrine manner [137], and in the rat B104 neuroblastoma cell line, the peptide also increased the proliferation of tumor cells [141]. By contrast, GAL exerted an antiproliferative effect via GAL_2_R in the human neuroblastoma SH-SY5Y cell line [12]. GAL_2_R mediated apoptosis in the latter cell line; however, the GAL antiproliferative potency was 100-fold higher in SH-SY5Y neuroblastoma cells overexpressing GAL_2_R than in SH-SY5Y neuroblastoma cells overexpressing GAL_1_R, suggesting that a high level of GAL_2_R is able to block tumor cell proliferation [12]. In this sense, GAL_2_R transfection into neuroblastoma SH-SY5Y cells inhibited cell proliferation and promoted a caspase-dependent apoptotic mechanism [12]. Finally, the expression of GAL has been reported in human paragangliomas [108,112,142] and the peptide was respectively found in 18% and 40% of carotid body or jugulo tympanic paragangliomas [108].

#### 3.1.4. Pituitary Tumor

GAL and the three GALRs have been observed in normal pituitary glands [30,36,145]. GAL was located in cells also containing growth hormone, prolactin, thyroid-stimulating hormone or adrenocorticotropic hormone (ACTH) [38,167]. GAL_1_R was the most abundant receptor observed in normal anterior pituitaries, followed by GAL_3_R, whereas GAL_2_R was not found [30]. In another study, GAL, GAL_1_R and GAL_2_R mRNAs were found in human pituitaries, but not GAL_3_R mRNA [145]. Estrogens increased GAL mRNA and peptide levels in the rat anterior pituitary [167].

GAL was detected in some, but not all, pituitary tumors [36] (Table 1). Importantly, GAL/GALR expression is related to the pituitary tumor stage. Human pituitary adenomas display an increased expression of GAL_1_R [145], while high levels of GAL_3_R have been reported in some patients who relapsed shortly after surgical intervention [145]. This suggests that GAL_3_R could be a marker for relapsing pituitary tumors and that GAL_3_R antagonists could be a therapeutic approach for the treatment of pituitary tumors [145]. GAL may promote pituitary cell proliferation and tumor development in an estrogen-dependent or independent manner. Thus, in the rat MtTW-10 pituitary tumor cell line, GAL mRNA levels highly increased after the administration of estradiol. These cells secreted GAL, a process that was blocked by somatostatin. In rats, a sexual dimorphism was observed in estrogen-induced anterior pituitary tumorigenesis (female tumors averaging twice the size of male tumors); this could be due to a differential expression of GAL [168].

GAL and prolactin coexist in lactotrophs [38,167]. In transgenic mice, the overexpression of GAL in these cells promoted the synthesis and release of prolactin favoring hyperprolactinemia; moreover, this study showed that pituitary GAL favored pituitary hyperplasia (especially lactotrophs) in an estrogen-dependent manner [169]. In fact, high estrogen levels promoted prolactin-secreting pituitary tumors, which in turn released GAL [148] and, in estrogen-induced prolactinomas, the expression of the GAL gene and the level and secretion of GAL increased in the rat anterior pituitary [147,160]. Thus, GAL acts as an autocrine/paracrine hormone, regulating the secretion of prolactin [160]. It has been reported that the synthetic progestin levonorgestrel reduced the pituitary growth by decreasing the expression of GAL [149]. 

The coexistence of growth hormone (GH) and GAL has been reported in somatotrophs [38,167]. GAL promoted the release of GH from normal rat pituitary cells, but the peptide blocked this release from rat somatotroph adenoma cells [158,159]. The GAL level was low in GH secreting adenomas, but the level of the peptide was high in corticotroph adenomas [102]. In humans, GAL increased the circulating level of GH and GH-producing tumors expressed GAL [157]. A high increase in both GAL mRNA and GAL expression/secretion was observed in GH-releasing hormone transgenic mouse (somatotroph hyperplasia) [147,160]. The data show that GAL plays an important role in pituitary hyperplasia mechanisms by promoting cell proliferation [38].

Most of the corticotroph adenomas express GAL [102]. In normal pituitaries, the co-existence of GAL and ACTH has been reported in corticotrophs and, in the same cells, GAL and ACTH were also co-expressed in nonfunctioning and functioning pituitary tumors [102,105]. GAL expression is related to smaller adenomas and better prognosis [102,105]. GAL has been observed in 84% of the corticotroph cell tumors associated with Cushing’s disease [103], although another study has reported that GAL did not play an important pathophysiological role in this disease because corticotroph adenomas can function irrespective of the presence of GAL [105]. GAL is secreted by human tumoral corticotrophs and responds to the corticotropin-releasing factor [135].

The expression of the GAL gene was blocked in thyrotroph adenomas; this means that GAL did not exert a stimulatory proliferative action on thyrotrophs [147]. Another study has shown that the synthesis of GAL was inhibited in thyrotroph adenomas [160].

#### 3.1.5. Small-Cell Lung Cancer

Small-cell lung cancer (SCLC) is a poorly differentiated neuroendocrine carcinoma [170]. Approximately, it accounts for 15% of all lung cancers, is very aggressive, and is the leading cause of cancer death worldwide in men [170,171,172].

GAL mediated, via GAL_2_R, the proliferation of SCLC cells [113,150,151,173] (Table 1). Ca^2+^-mobilizing peptides (e.g., GAL, neurotensin, cholecystokinin) promoted the growth of SCLC cells through autocrine and paracrine mechanisms [150]. This finding suggests that broad spectrum antagonists directed against multiple Ca^2+^-mobilizing receptors could exert a therapeutic antitumor action and, in fact, these antagonists inhibited SCLC cell growth [152]. In H69 and H510 SCLC cell lines, GAL increased the formation of inositol phosphate and the intracellular level of Ca^2+^, and the peptide also promoted the growth of both cell lines, which was dependent on the concentration of GAL [151]. GAL, mediated by the p42MAPK pathway dependent on the activity of PKC, promoted the growth of SCLC cells, which was blocked with PKC inhibitors [153,154]. SBC-3A SCLC cells release pre-pro-GAL precursors, but not active peptides; however, extracts from mouse SBC-3A tumors contained pre-pro-GAL precursors and GAL1-20 (a cleaved lower-molecular mass of GAL) [156]. This means that pre-pro-GAL precursors were extracellularly processed to GAL1-20 and, in fact, it was demonstrated that the protease plasmin (present in SBC-3A tumors) was responsible for the processing of the pre-pro-GAL precursors to GAL1-20 [155,156]. GAL promoted the release of the promatrix metalloproteinase-2/9 from SBC-3A SCLC cells [156], and SCLC cells produced and released GAL, which exerted, via GAL_2_R, a mitogenic action on these cells by activating Gq, Gi and G12 G proteins [88]. Thus, GAL activates multiple signals through the G12/Rho pathway and the Gq phospholipase C/calcium sequence and also promotes Ca^2+^ mobilization [88].

### 3.2. Galanin and Gastric Cancer

In nerve cells, the galaninergic system plays an important role in tumor development. In human stomach samples, obtained from the vicinity of invasive cancer cells, neurons located in the myenteric plexus showed a high expression of both caspases 3 and 8, but a low expression of GAL [45,174] (Table 2). In carcinoma-affected regions of the human stomach, an increase of the GAL-immunoreactive fibers in the longitudinal muscle layer, lamina muscularis mucosae and in the vicinity of the neoplastic proliferation was observed; thus, carcinoma invasion affected GAL stomach wall innervation [175]. In patients suffering from gastric cancer, lower levels of GAL were observed in pre-operative samples (and in plasma) when compared with those found in post-operative samples obtained from the same patients or from samples of healthy donors [176]. Moreover, the levels of GAL/GAL_1_R were lower in gastric cancer tissues compared with those found in adjacent regions; however, the GAL_2_R/GAL_3_R levels did not change [176]. The low level of GAL could be used as a biomarker in gastric cancer and, importantly, in these patients (pre-operative samples), the GAL protein/mRNA levels have been related to tumor size, tumor node metastasis stage and lymph node metastasis [176].

A prolonged administration of GAL (4 µg/kg) blocked gastric carcinogenesis by inhibiting the proliferation of antral epithelial cells [13]. Human gastric cancer cells (AGS, KATOIII, SNU-638, SNU-601, SNU-1) showed a low endogenous GAL expression, which was restored with a demethylating agent (5-aza-2′-deoxycytidine) [177]. In addition, the hypermethylation of GAL impaired its tumor suppressor action in gastric cancer, and the exogenous GAL expression in silenced cells promoted a decrease in phosphorylated Akt expression and apoptosis [177]. This means that the downregulation of GAL in gastric tumor cells was due to an epigenetic inactivation. Finally, GAL decreased the proliferation of human gastric cancer cells in vitro [178].

### 3.3. Galanin and Colorectal Cancer

Colorectal cancer (CRC), the third most prevalent cancer worldwide, is an invasive tumor process due to the proliferation of epithelial cells that acquire a neoplastic phenotype [8]. This process is known as epithelial-to-mesenchymal transition, in which epithelial cells lose many morphological and functional characteristics (e.g., shape, cell polarity, intercellular junctions) [8]. Tumor cells digest the extracellular matrix of the intestine wall, activating growth factors that promote cell proliferation, the blockade of apoptotic mechanisms and also favor the spreading of cancer cells [8]. Then, the invasion of cancer cells destroys the enteric nervous system, leading to the atrophy of the submucosal/myenteric plexuses. The galaninergic system is involved in colon cancer [106,117,121] (Table 2); thus, for example, the siRNA-mediated silencing of the GAL gene reduced both invasive and proliferative potential in CRC cells [117].

CRC tissues showed higher GAL levels than the corresponding non-tumor tissues [106,117,179,180], and human colon cancer cell lines (LOVO, HCT15, SW480, SW620) showed higher levels of GAL than those found in non-colon cancer cell lines [106]. In blood samples of CRC patients, an increased concentration of GAL (2.4 times higher) has been reported [179]. GAL mRNA is overexpressed in CRC and its level has been related to adenocarcinoma size/stage and a correlation between shorter disease-free survival of early-stage CRC patients and high expression of GAL has been reported [7,106,121]. In CRC patients, a high GAL expression was related to tumor recurrence, and CRC patients (stage II) who showed a high GAL expression had a poorer prognosis than those showing a low expression of the peptide [180]. In addition, a relationship between a high GAL expression and the spread of cancer stem cells (metastasis) has also been reported in CRC (stage II) [180]. However, an association between survival and GAL expression was not observed in CRC patients (stage III) [180]. The data show that the expression of GAL is related to CRC aggressive behavior and it seems that CRC cells showing a high GAL expression are more malignant and are also involved in the recurrence of the tumor [180]. However, a recent study has shown that GAL downregulation is correlated with advanced CRC stages in northern African individuals and it is linked to autophagy, cell cycle and division, immune system response and the transcriptional regulation of TP53 [183]. Compared to epithelial cells of the large intestine, a stronger immunoreactivity for GAL_1_R/GAL_3_R was observed in CRC cells and it has been reported that the high expression of GAL_3_R in CRC tissue was associated with a better prognosis and longer survival of CRC patients; this means that GAL_3_R is a prognostic factor for these patients [184].

The number of neurons containing GAL was higher in CRC patients than in those showing a healthy intestine, and an increased GAL gene/protein expression was observed in CRC tissues [106,179]. Compared to control individuals, a higher percentage of neurons containing GAL was reported in the myenteric plexus of CRC patients; however, no change was observed regarding the density of the immunoreactive fibers containing GAL located in the myenteric and submucosal plexuses [121]. The number of neurons containing GAL also increased in the tissue regions located close to CRC; thus, the release of GAL from these neurons could block apoptotic mechanisms favoring tumor cell survival and proliferation [8,185]. In fact, GAL promoted CRC cell proliferation and improved cell survival [8], and then cancer cell invasiveness increased and tumor development was accelerated. In another study, CRC tumor samples were collected as well as colon wall tissues located close to and distant from the neoplastic tissue: a high GAL immunoreactivity was observed in myenteric/submucosal plexuses, intestinal epithelium and cancer cells, whereas the lowest GAL level was found in the muscular layer located distant from the tumor [179]. The author concluded that GAL could be a potential biomarker for CRC tumors.

GAL_1_R is mainly expressed in the human colon. The silencing of this receptor or GAL promoted apoptosis in drug-sensitive/resistant cell lines and enhanced the effects mediated by chemotherapy; thus, GAL_1_R regulates drug resistance [117]. The GAL_2_R gene has been suggested in CRC as a chemosensitive methylation candidate to bevacizumab, since HCT116 CRC cells overexpressing GAL_2_R were more chemosensitive to the monoclonal antibody than control cells [181]. GAL_1_R silencing promoted a downregulation of the FLIPL-like inhibitory protein long form (FLIPL, a caspase 8 inhibitor), meaning this inhibitor is a key downstream effector of the anti-apoptotic signaling mediated by GAL/GAL_1_R [117]. Thus, the downregulation of the inhibitor favors the induction of caspase 8-dependent apoptotic mechanisms. Finally, GAL decreased the incidence of colon tumors in rats and it seems that this effect was due to the inhibitory action exerted by GAL on cancer cell proliferative mechanisms [182].

### 3.4. Galanin and Head and Neck Squamous Cell Carcinoma

Head and neck squamous cell carcinoma arises from mucosal surfaces of the head and neck [186] (Table 3). Perineural invasion (PNI), a mechanism of tumor dissemination via nerves, predicts poor survival in some cancers including head and neck squamous cell carcinoma (HNSCC), pancreatic cancer, stomach cancer and colon cancer, and is a sign of cancer cell invasion and metastasis [187]. An interaction between nerves and tumor cells occurs in PNI. PNI, mediated by molecular signals, promoted neuritogenesis and the survival, proliferation and invasion of tumor cells [84,188,189,190]. These cells are attracted to nerves and communicate with them. GAL (released from nerves) exerted a nerve–tumor crosstalk by activating GAL_2_R expressed in tumor cells and by inducing NFATC2-mediated transcription of cyclooxygenase-2 and GAL; then, GAL released from tumor cells promoted neuritogenesis, favoring PNI [84].

The promoter methylation status of the peptide-encoding gene GAL was studied in HNSCC samples; methylation was observed in 20% of them [192]. The authors showed that the methylation status of some peptide-encoding genes, including GAL, was related to survival and recurrence in HNSCC, and they also suggested that methylation changes could be a possible molecular marker for HNSCC risk/prognosis. In fact, poor survival has been associated with the methylation of GAL/GAL_1_R genes, and a hypermethylation promoted the inactivation of GAL/GAL_1_R/GAL_2_R genes [195]. The GAL_1_R gene promoter is widely hypermethylated in HNSCC (cell lines, primary tumor); this is related to reduced GAL_1_R expression, which can be restored by treating with a histone deacetylase inhibitor (trichostatin A) or with a methyltransferase inhibitor (5-azacytodine) [191,198]. This is important, since the methylation of the GAL_1_R gene promoter has been related to HNSCC carcinogenesis [193]. GAL_1_R/GAL_2_R hypermethylation has been associated with a higher recurrence rate and reduced disease-free survival [194,200]. GAL/GALR epigenetic variants are excellent markers for the prognosis prediction of patients suffering from HNSCC [193,194]; thus, the GAL_1_R methylation status could be a biomarker for predicting HNSCC clinical outcomes. Importantly, because methylation suppresses GAL/GALRs expression in some tumors and because GAL/GAL_1_R act as tumor suppressors (see below) [177], these findings suggest that the methylation-based suppression of GAL/GALRs eliminates the expression of a tumor-suppressive pathway.

A high level of GAL has been detected in HNSCC [120]; GAL/GAL_1_R blocked human oral tumor cell proliferation [177], and GAL_1_R inhibited the proliferation of keratinocytes (malignant and immortalized) by blocking the MAPK pathway [123]. Thus, GAL_1_R acts as a tumor suppressor gene, which is frequently silenced in HNSCC [177,198]; in fact, in some HNSCC cell lines, the expression of GAL_1_R is absent [198]. GAL_1_R blocked the proliferation of tumor cells through the activation of ERK1/2 and cyclin-dependent kinase inhibitors, leading to cell-cycle arrest (regulating cell cycle control proteins such as cyclin D1, p57, p27) [123,196,198,200]. Moreover, the re-expression of GAL_1_R in GAL_1_R/GAL_2_R-negative HNSCC cells also suppressed tumor cell proliferation through ERK1/2-mediated actions on cyclin-dependent kinase inhibitors and cyclin D1 [113]. The overexpression of GAL_2_R in HNSCC cell lines favored the survival and proliferation of these cells by activating respectively the PI3K/Akt and MAPK/ERK-dependent pathways [122]. Rap1 (a Ras-like signaling protein) is involved in HNSCC progression [122], and GAL_2_R activated rap1B (small-GTP protein) favoring a p38-mediated inactivation of the mRNA binding protein tristetraprolin, which inhibited the production of many pro-inflammatory cytokines. This means that GAL_2_R-p38-mediated cytokine production could be a therapeutic target against HNSCC, since p-38 inhibitors are currently used in clinical practice. In HNSCC, GAL_2_R promotes tumor angiogenesis by enhancing the secretion of cytokines (vascular endothelial growth factor, interleukin-6) via the p38-MAPK-mediated inhibition of tristetraprolin [201]. By contrast, GAL_2_R exerted an antitumor effect by inducing cell-cycle arrest and apoptotic mechanisms (caspase 3-dependent) [190] and this means that the activation of these mechanisms could exert a beneficial therapeutic action against HNSCC. The proliferative or antiproliferative actions mediated by GAL_2_R in HNSCC could be explained by the signaling pathways activated depending on the coupled G protein type. Moreover, GAL_2_R transfection into human HNSCC cells suppressed cell proliferation [113,197] and the re-expression of GAL_2_R blocked HNSCC cell proliferation (showing mutant p53) [113]. Importantly, apoptotic mechanisms via the activation of GAL_1_R by GAL have not been reported, and in HNSCC cells, GAL_1_R/GAL_2_R are suppressor tumors in a p53-independent manner [11]. GAL_2_R mediated apoptotic mechanisms (caspase-independent) in HEp-2 cells by downregulating ERK1/2 and inducing Bim (a pro-apoptotic Bcl-2 protein) [199]. Although the receptors tend to be tumor suppressive, it has recently been reported that GAL released by HNSCC cells exerted a pro-tumoral and immune-suppressive effect and data from the Cancer Genome Atlas have shown that a reduced overall survival of HNSCC patients was correlated with a high expression of GAL [202].

### 3.5. Galanin and Glioma

The GAL/GALR system has been described in glioma [30,118] in which the most abundant receptor observed was GAL_1_R, followed by GAL_3_R; GAL_2_R was not found (astrocytic/oligodendroglia tumors) [30] (Table 4). A reduced level of GAL has been observed in the cerebrospinal fluid of patients with glioblastoma [203], and regarding the expressions of GAL and GAL_3_R, no correlation with oligodendroglial, astrocytic and mixed neural–glial tumors was reported [30]. Moreover, no correlation was observed between the proliferative activity and GAL/GAL binding levels [118]. However, the high-grade glioma (WHO grade IV) has been related to the expression of GAL_3_R [30]. GAL has been reported in gliosarcoma and glioblastoma multiforme [118]; in the latter, the most abundant receptor found was GAL_1_R, followed by GAL_3_R and GAL_2_R [118]. In glioma, endothelial and immune (e.g., macrophages, neutrophils) cells expressed GAL_3_R, but GAL_1_R/GAL_2_R were not observed around the blood vessels [30]. This means that tumor-associated cells are involved in tumor microenvironment homeostasis. Glioma-associated macrophages (GAMs) are involved in tumor progression; although macrophages produce/secrete GAL, GAMs do not express GAL, but express GAL_3_R, and this means that GAL could regulate the activity of GAMs [59,204].

GAL blocked, through GAL_1_R, the proliferation of human glioma cell lines (U251, T98G) and tumor growth in nude mice [205]. The authors reported that GAL did not exert cytotoxic/apoptotic effects and that the blocking actions exerted by GAL were due to the activation of the ERK1/2 signal.

### 3.6. Galanin and Other Cancers

Although the expressions of GAL and pre-pro-GAL mRNA have been reported in breast cancer, it has been suggested that the GALN gene (which encodes the pre-pro-GAL protein) is an unlikely candidate oncogene in breast tumors because an increase in pre-pro-GAL mRNA expression with GALN amplification was not observed [101,206] (Table 5). Many nerve fibers containing GAL have been reported in cardiac and esophageal carcinomas [207]; these fibers contacted closely with cancer cells, including those encircling tumor cells. In this study, GAL favored the extension of processes by dorsal root ganglion neurons, but the action of the peptide on tumor cells is currently unknown [207]. GAL_1_R DNA methylation is among the most epigenetic molecular alterations in endometrial cancer; this methylation indicates malignancy with a high degree of sensitivity and specificity [208]. The methylation of the GAL_1_R gene in bladder cancer has been involved in the prognosis of the disease, but the role played by the galaninergic system in this cancer is currently unknown [209].

It has been suggested that GAL_1_R/GAL_2_R are therapeutic targets and prognostic factors in salivary duct carcinoma [210]. GAL_1_R/GAL_2_R methylation rates were higher in salivary duct carcinomas than in normal tissues, and these rates were correlated with a decrease in overall survival. The expression of GAL has been reported in melanoma, and human Bowes melanoma cells expressed GAL_1_R [119,213]. In the latter case, a biphasic response (increase of the extracellular acidification rate followed by a decrease below the basal level) was found after the activation of the receptor, being the magnitude of the response depending on the concentration of GAL [213]. GAL blocked pancreatic carcinogenesis in rats, and this was related to the inhibition of the activity of the sympathetic nervous system [212]. The latter study demonstrated that animals treated with GAL showed a lesser number of pancreatic adenocarcinomas than control animals and that GAL decreased the pancreatic level of norepinephrine. By contrast, GAL promoted the proliferation of SW1990 human pancreatic cancer cells in vitro [211]. These contradictory findings may be due to the fact that the former experiment was performed in vivo [212].

## 4. The Galaninergic System and Cancer: Signaling Pathways

**Figure 4:**
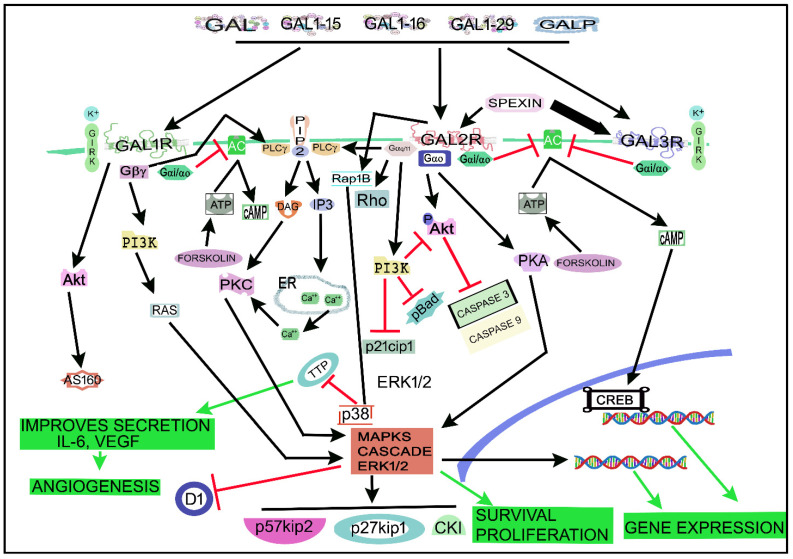
Main signaling pathways in which the galaninergic system is involved. Black arrows indicate activation pathways, inverted red “T” indicates blockade/suppression, green arrows mean final results. AC, adenylate cyclase; Akt, Akt serine/threonine kinase family (also called PKB); AS160, Akt substrate of 160 kDa; ATP, adenosine triphosphate; Ca^2+^, calcium ion; cAMP, cyclic adenosine monophosphate; CKI, cyclin-dependent kinase inhibitor 1; CREB, cAMP regulatory element-binding protein; D1, a cyclin protein; DAG, diacylglycerol; ER, endoplasmic reticulum; FORSKOLIN, enzyme that produces cyclic adenosine monophosphate; GAL, galanin; GAL1-15 fragment, galanin 1–15 fragment; GAL1-16, galanin 1–16 fragment; GAL1-29, galanin 1–29 fragment; GAL_1_R, galanin receptor 1; GAL_2_R, galanin receptor 2; GAL_3_R, galanin receptor 3; GALP: GAL-like peptide; GIRK, G protein-coupled inwardly-rectifying potassium; Gα/11, G protein alpha subunit (11); Gαi/αo, G protein alpha i/o subunits; Gαo, G protein alpha subunit (o); Gβγ, G protein beta-gamma subunit; IL-6, interleukin 6; IP3, inositol triphosphate; K^+^, potassium ion; MAPK, mitogen-activated protein kinases cascade; p21cip1, a cyclin-dependent kinase inhibitor; p27kip1, cell-cycle control protein; p38, a class of mitogen-activated protein kinase; p57kip2, cell-cycle control protein; pAkt, phosphorylated Akt; pBad, phosphorylated BAD forms (induces apoptosis by inhibiting antiapoptotic BCL-2 family members); PI3K, phosphatidylinositol 3-kinase; PIP2, phosphatidylinositol bisphosphate; PIP2, phosphatidylinositol, 4, 5-bisphosphate; PKA, protein kinase A; PKC, protein kinase C; PLC, phospholipase C; Rap1B, Ras-related protein Rap-1b; Ras, rat sarcoma virus (a small GTPase); Rho, a family of small signaling G proteins (a subfamily of the Ras superfamily); TTP, tristetraprolin; VEGF, vascular endothelial growth factor.

Figure 4 shows the main signaling pathways in which the galaninergic system is involved. A GAL/GALR signaling network map focused on the signaling cascades regulated by the galaninergic system has recently been published [87]. GALRs (via PKC) activate the rat sarcoma virus (Ras, a small GTPase)/MAPK/ERK pathway by increasing the intracellular Ca^2+^ concentration [8]. The galaninergic system activates many signal transduction pathways depending on the coupled G protein type: GAL_1_R/GAL_3_R, mainly coupled to Gi/o, decrease the cAMP level and inactivate PKA, whereas GAL_2_R, preferably coupled to Gq/11 mobilizing intracellular Ca^2+^, promotes (via PKC) the activation of cell survival (via Akt or PKB) and MAPK1/MAPK3-dependent cell proliferation pathways [17,30,87]. GAL_1_R can also be coupled to Gβγ- and/or Gi-signaling pathways and then the activation of MAPKs occurs in a Ras/Raf-dependent manner [17,25,87]. GAL_1_R activation also favors the Akt/Akt substrate of the 160 kDa (AS160) cascade [87], regulates GIRK channels [4,77] and activates the ERK1/2 signal through the Gα/i subunit and not via the PI3K pathway linked to the Gβγ subunit [196]. GAL_1_R induces cell-cycle control proteins (p27kip1, p57kip2) and suppresses cyclin D1 in cancer cells [20]. GAL_2_R, mainly coupled to Gq/11, mediated the activation of PLC and small GTPase proteins in the Rho family [87]. PLC converted phosphatidylinositol, 4, 5-bisphosphate (PIP2) into diacylglycerol (DAG) and inositol triphosphate (IP3), which mediated PKC activation and increased the intracellular concentration of Ca^2+^ [77]. GAL_2_R activated the small GTPase protein Rho A in SCLC cells, suggesting the coupling to G12/13 [20]. GAL_2_R inhibited the production of cAMP, meaning that the receptor was coupled to Gi protein [83]. GAL_2_R decreased cofilin activation and Rho and Cdc42 GTPase activity [20]. In tumor cells, GAL_2_R activated the MAPK/ERK pathway in a PKC manner, meaning that GAL_2_R was coupled to a Go protein [20]. GAL_2_R regulated cell-cycle control proteins (p27kip1, p57kip2) and cyclin D1 and promoted apoptosis (caspase 3-dependent) in HNSCC cells [26]. GAL_2_R decreased the expression of p21cip1, phosphorylated BAD forms (pBad) and phosphorylated Akt (pAkt), downstream of the Gq11/PI3K pathway [26]. The GAL-mediated Akt pathway blocked the activity of caspases 3 and 9, whereas the GAL_2_R-mediated apoptosis in tumor cells was induced by the activation of the pro-apoptotic Bcl-2 protein Bim, through a mechanism independent of caspase [20]. GAL_3_R, involved in inward potassium ion (K^+^) currents, is coupled to the Gi/o signaling pathway and its activation favored the inhibition of cAMP and AC altering CREB phosphorylation [17,87,90]. GAL opened adenosine triphosphate (ATP)-sensitive K^+^ channels and hyperpolarized cell membranes in the rat RINm5F insulinoma cell line [214], and the peptide blocked the activity of AC and the secretion of insulin via the interaction with Gαi1, Gαi2 and Gαi3 proteins [53,215]. C7 peptide (GAL1-13-spantide amide), a GAL receptor antagonist, blocked hepatocellular carcinoma metastasis by targeting the hepatocyte growth factor/c-mesenchymal–epithelial transition receptor axis signaling pathway [216]. C7 inhibited the migration and invasion of tumor cells by blocking the phosphorylation of Akt and ERK1/2 [216].

The interaction between GAL_1_R/GAL_2_R-5-hydroxytryptamine 1A receptor heteromer (a macromolecular complex formed by at least two different receptor units) promoted conformational changes in GAL recognition sites, altering the binding affinity of GAL [100]. In this sense, conformational changes in the GAL_1_R/GAL_2_R complex favored a higher affinity of GAL_1_R for GAL1-15 than for GAL, increasing Gi/o-mediated signaling and decreasing AC activity and CREB levels [98]. In addition, GAL_3_R heteromerization with other peptide receptors or other GALRs has been suggested [20].

## 5. Therapeutic Strategies 

Peptides play an important role in cancer; the in-depth knowledge of the functions mediated by these substances is an emerging and promising line of research that could lead to new clinical applications in oncology. One line of research could be the use of peptides coupled to cytotoxic agents to exert an antitumor action, and another, the use of peptide receptor antagonists or agonists. In the case of GAL, GALR antagonists or agonists could be used as antitumor treatments according to the different signaling pathways and actions mediated by GALRs. GALR antagonists have been administered for the treatment of food intake disorders, anxiety, depression and Alzheimer’s disease, whereas GALR agonists have been used for the treatment of chronic pain [18,93]. It has also been reported that SNAP 37889, a non-peptidergic GAL_3_R antagonist, promoted apoptosis in promyelocytic leukemia cells expressing GAL_2_R [217].

In vitro and in vivo experiments using human gastric cancer cell lines have been performed to study the antitumor action of a triple treatment with GAL, serotonin and octreotide (an octapeptide that mimics the actions mediated by somatostatin) [178]. Treatment with one compound or with a double/triple combination decreased cell proliferation and viability in vitro, and tumor volume/weight was reduced in vivo after the triple treatment. However, this reduction was not due to apoptosis or cell proliferation inhibition; thus, other unknown mechanisms were involved [178]. In experimental animals, implanted human colon cancer cells were treated with the triple treatment (octreotide, serotonin and GAL were administered subcutaneously or intraperitoneally) [218,219,220]: tumor volume/weight, number of viable cells, proliferation index and tumor vascularization decreased, whereas the apoptotic index increased. In nude mice implanted with colonic adenocarcinoma cells and treated with the triple treatment, the tumor volume decreased and the apoptotic index and volume density of the tumor necrotic tissue increased [221]. The triple therapy did not show any apparent side effects [222]. Low concentrations of GAL, somatostatin and serotonin have been reported in CRC patients and treatment with GAL alone showed an important decrease in the number of tumor blood vessels [223]. Comparing the administration of one, two or three compounds, the antitumor effect was higher when the three compounds (GAL, serotonin, octreotide) were administered [224]. Importantly, the antitumor effect promoted by the triple therapy was comparable to the treatment with 5-fluorouracil/leucovorin, a chemotherapeutic agent used for CRC treatment [225]. Triple treatment has a better safety profile and, hence, it is a potential therapeutic strategy against CRC [226], but more preclinical and clinical studies are needed to confirm its beneficial use in clinical practice.

Peptide analogs have been used as an antiproliferative strategy and promising results targeting peptidergic systems have been reported; accordingly, GAL analogs could be tested in tumors expressing certain GALRs. The half-life of GAL in plasma is about 5 min, but the half-life of synthetic GAL (e.g., GAL1-29, GAL1-16) is 60–120 min. GAL1-16 was synthesized as a free carboxylic acid, whereas GAL1-29 was synthesized with a C-terminal amide corresponding to the endogenous peptide; the data mean that analogs showing an increased half-life are required for a therapeutic application of the peptide [20]. Ligand specificity/selectivity must be understood in-depth at GALRs to understand the molecular interactions that occur in these mechanisms and to develop drug-design studies. It is important to note that, currently, there are few experiments focused on the antitumor activity mediated by GALR antagonists or agonists [217]; this is a promising research field that must be developed immediately, since many in vitro and in vivo experiments are required to fully demonstrate the anticancer properties of GALR antagonists or agonists. Moreover, radiolabeled cytotoxic agents linked to peptides have been used for therapeutic applications (e.g., neurotensinergic system, substance P/neurokinin-1 receptor system) [9,227,228]. Again, this line of research (peptide and non-peptide ligands as radiopharmaceuticals) must be developed as targeted radionuclide cancer therapy in tumors expressing GALRs because it could serve to demonstrate the potential use of GALR agonists or antagonists in nuclear medicine for the diagnosis/treatment of GALR-positive tumors. Thus, it is important to know whether GALRs are molecular targets to radiosensitize cancer cells.

## 6. Discussion

**Figure 5:**
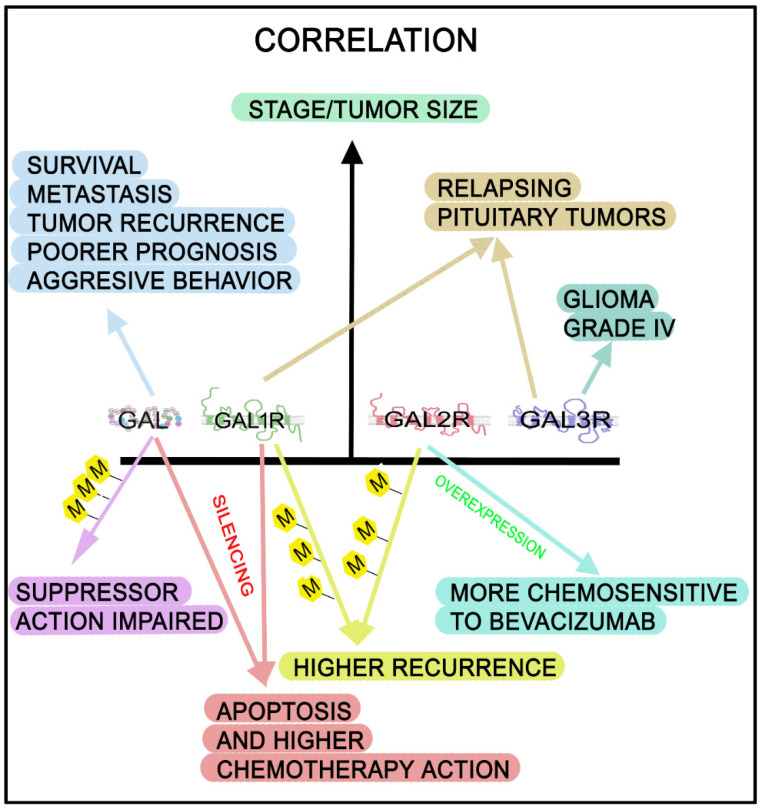
The galaninergic system has been correlated with survival, metastasis, tumor recurrence and poorer prognosis. M: methylation; M,M,M: hypermethylation.

The potential antitumor clinical application of GALR ligands (GALR antagonists or agonists) has unfortunately been neglected by the scientific community and the pharmaceutical industry. However, the expression of the galaninergic system could be used for the diagnosis, treatment and prognosis of tumors [30,36,39,101,102,103,104,105,106,107,108,109,110,111,112], and this system has also been correlated with tumor stage/subtypes (Figure 5). Stage/tumor size has been related to the level of GAL mRNA in colon cancer: the higher the GAL expression, the shorter the disease-free survival [30,101,106]. In neuroblastic tumors, a low level of GAL binding sites has been correlated with survival and GAL/GALR expressions have been related to tumor differentiation stages [136,137]. GAL expression has also been related to smaller adenomas and better prognosis [102,105]; the low level of GAL has been suggested for use as a biomarker in gastric cancer, and the level of GAL has been related to tumor size, tumor node metastasis stage and lymph node metastasis in patients suffering from gastric cancer [176]. A correlation between the shorter disease-free survival of early-stage CRC patients and the higher expression of GAL has also been reported [7,106,121]; the expression of GAL has been related to the aggressive behavior of CRC, and a relationship between a high GAL expression and metastasis has been observed in CRC [180]. 

Some peptidergic systems (e.g., substance P/neurokinin-1 receptor system, neurotensinergic system) exclusively promote the proliferation of tumor cells; however, GAL, via different GALRs, exerts a tumor cell proliferative action, but also the peptide suppresses the development of tumors [8,11,12,13,102,122,157]. This is an important characteristic of the galaninergic system that opens the door to a double potential therapeutic strategy using GALR agonists or antagonists. For this reason, it is crucial to determine which are the GALRs involved in cancer to develop specific antitumor ligands and drug-design studies; this is a line of research yet to be explored. The expression of GAL_1_R has been demonstrated in insulinoma cells [32,162]; however, the proliferative or antiproliferative actions mediated by GAL on these cells are currently unknown. Importantly, GAL’s antiproliferative potency was much higher in GAL_2_R-expressing cells than in those expressing GAL_1_R, meaning that a high level of GAL_2_R could block cancer cell proliferation [12]. The expression of GAL_3_R could be used as a marker for relapsing pituitary tumors and GAL_3_R antagonists could also be used to treat these tumors [145]; this must be confirmed. The GAL gene expression was blocked in thyrotroph adenomas [147,160]; these inhibitory mechanisms must be studied in-depth, since they could be useful to develop antitumor strategies. Another important point is to understand the role played by GAL in sexual dimorphism in estrogen-induced anterior pituitary tumorigenesis, since female tumors average twice the size of male tumors [168]. GAL, via GAL_2_R, promoted the proliferation of SCLC cells through an autocrine manner [88,113,150,151,173]; however, it is currently unknown whether or not GAL_2_R antagonists exert an antitumor action against SCLC cells. GAL inhibited the proliferation of glioma cells and tumor growth via GAL_1_R [205]; a reduced level of GAL was observed in the cerebrospinal fluid of patients with glioblastoma [203], and GAL_3_R expression has been related to high-grade glioma [30]: the line of research on glioma must be developed in the future. In other cancers, the galaninergic system must be better studied, since the current data are fragmentary. Thus, it must be confirmed whether or not GAL plays an important role in breast cancer, and the proliferative/antiproliferative action of the peptide in bladder cancer, melanoma and cardiac and esophageal carcinomas must be investigated in-depth. Confirmation is also required as to whether GAL_1_R/GAL_2_R are therapeutic targets and prognostic factors in salivary duct carcinoma, as well as whether GAL_1_R DNA methylation indicates malignancy or not in endometrial cancer. Finally, the dual role of GAL as a proliferative and antiproliferative agent must be clarified in pancreatic carcinogenesis.

**Figure 6:**
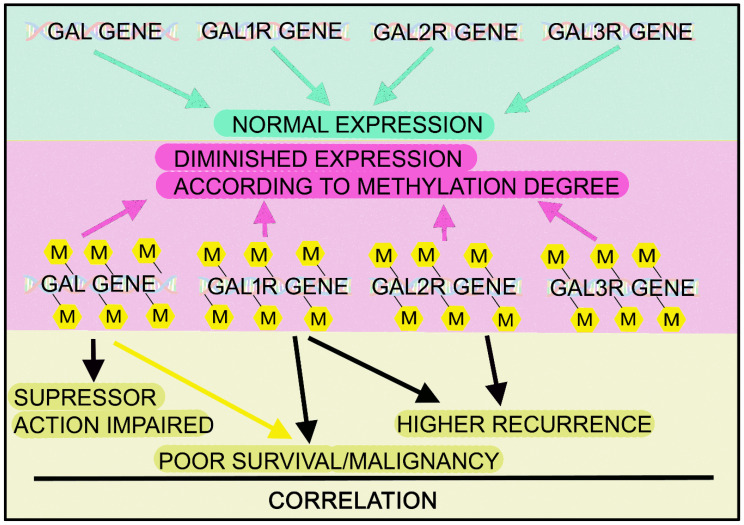
Effects of the epigenetic alterations in the galaninergic system: higher recurrence, malignancy and poor survival. Methylation (M)/hypermethylation (M,M,M) of GAL/GALR genes.

Epigenetic mechanisms regulate the galaninergic system and play a crucial role in tumor development (Figure 6). GAL downregulation favored tumor development in gastric cancer, which was due to an epigenetic inactivation, since the hypermethylation of GAL impaired its tumor-suppressive action [177]. Poor survival has been associated with the methylation of GAL/GAL_1_R genes in HNSCC and it has been reported that hypermethylation promoted the inactivation of GAL/GAL_1_R/GAL_2_R genes [195]. Thus, methylation changes could be a possible molecular marker for HNSCC risk/prognosis, since the methylation of the GALR gene promoter has been related to HNSCC carcinogenesis [193], and GAL_1_R/GAL_2_R hypermethylation has been associated with higher recurrence rates and reduced disease-free survival [194,200]. The GAL_1_R methylation status could be a biomarker for predicting HNSCC clinical outcomes. However, more studies must be performed to confirm whether GAL_1_R/GAL_2_R are potential therapeutic targets and prognostic factors.

Many of the proliferative and antiproliferative actions mediated by GAL on tumor cells could be explained by the signal transduction pathways depending on the coupled G protein type [17,30,77,87,196]. These actions could be also explained by GALR heteromer complexes, formed by GALRs with each other or with other types of G protein-coupled receptors, that promote conformational changes in GAL recognition sites, altering the binding affinity of GAL and favoring a certain signaling pathway [99]. This line of research must be investigated in-depth and it will serve to identify potential antitumor targets against the galaninergic system. For example, the blockade of signaling pathways common to several peptides could be an effective antitumor strategy as well as the development of broad-spectrum antagonists. Detailed studies on the antitumor effects of GAL agonists or antagonists have yet to be conducted in many types of cancer; thus, to obtain a detailed understanding of the different distribution patterns of GALRs and the different signaling pathways involved in tumor cells will help to identify the antiproliferative or proliferative actions played by these receptors and to develop new antitumor strategies. The use of an antitumor triple therapy (GAL, serotonin, octreotide) has been tested with good results against some tumors [219,220,223], but for unknown reasons, these investigations were not continued. Additional experiments are required to confirm the anticancer actions exerted by the three compounds. Moreover, two important lines of research must be developed: (1) the use of GAL analogs in tumors expressing certain GALRs; and (2) targeted radionuclide cancer therapy: the use of GAL and non-peptide ligands as radiopharmaceuticals for the diagnosis/treatment of GALR-positive tumors. Because GALRs play a crucial role in certain tumors, it is important to determine whether GALRs are involved in the viability of cancer cells, as has previously been demonstrated in tumor cells for the neurokinin-1 receptor [10] Moreover, the possible tumor and/or antitumor actions mediated by GAL fragments and other members of the GAL family of peptides such as GALP, spexin, alarin and GMAP must also be studied, since most of the studies in this field have focused on the entire molecule of GAL. Finally, it is important to note that the expression of peptides (e.g., neurotensin) has been reported in fetal tissues, but not in adult organs [9]. The authors suggested that the expression of peptides in these organs could be related to a malignant transformation, probably due to the presence of stem cells expressing peptides. This also suggests that a reversal to the fetal expression pattern occurred. This is an interesting issue that must be studied in the galaninergic system.

## 7. Conclusions

**Figure 7:**
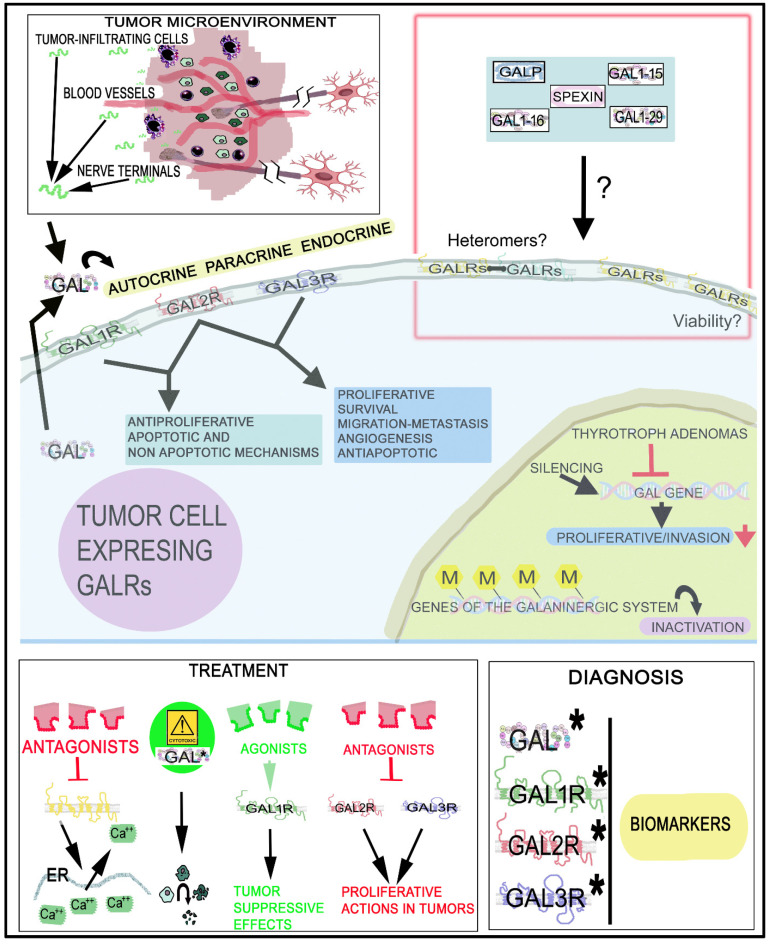
Involvement of the GAL/GALR system in cancer, diagnosis and treatment. GAL_1_R/GAL_2_R mediate an antiproliferative effect, whereas GAL_2_R/GAL_3_R promote a proliferative action on tumor cells. GAL originates from tumor cells, tumor-infiltrating cells and nerve cells. Circulating GAL can also bind to GALRs. ↑: increase; ↓: decrease; ?: mechanisms that must be investigated (presence/functions of heteromers in tumor cells, involvement of GALRs in the viability of cancer cells and involvement of GAL fragments and other peptides belonging to the GAL family of peptides in cancer). *, biomarkers; M: methylation.

The galaninergic system is involved in tumorigenesis, invasion and migration and has been correlated with tumor stage/subtypes and metastasis and, in this system, epigenetic mechanisms have been related with carcinogenesis and recurrence rates. GALRs play a crucial role in cancer and their specific actions must be clearly understood in many tumor types because GALRs mediate different signal transduction pathways and actions depending on the tumor cell type and the particular G protein involved. GALRs could be used as a therapeutic target and diagnostic marker for the treatment, prognosis and surgical outcome in certain tumors. Different from other peptidergic systems, the galaninergic system exerts a proliferative action on tumor cells, but GAL also suppresses the development of tumors (Table 6). Thus, in-depth studies using GALRs agonists or antagonists as antitumor agents must be conducted to search for therapeutic strategies (alone or in combination with chemotherapy/radiotherapy) against tumor development. The involvement of the galaninergic system in cancer is a line of research that has been abandoned, but it must be re-opened and developed in the future. Additional studies must be carried out, for example, on the use of GALR agonists/antagonists as antitumor agents, the activation of signaling transduction pathways, the involvement of heteromers, targeted radionuclide cancer therapy and the viability of GALRs. This knowledge is crucial to establish future potential clinical antitumor applications, although unfortunately, the pharmaceutical industry has generally had no interest in this line of research; however, the data reported here suggest that the galaninergic system is a promising target for the treatment of tumors (Figure 7).

## Figures and Tables

**Table 1:** Involvement of the galaninergic system in neuroendocrine tumors.

Cancer	Actions/Presence	References
Corticotroph adenoma Human	- High GAL expression (RIA)	[102]
	- GAL in 84% of tumors (IH)	[103]
	- GAL expression: smaller adenomas and better prognosis (IH)	[105]
	- GAL release and responded to corticotropin-releasing factor	[135]
Ganglioneuroma Human	- No correlation between prognosis/tumor markers and GAL level (RIA)	[136]
	- GAL1R/GAL3R immunoreactivity decrease (IH)	[137]
Insulinoma Rat Rin14B cell line	- GAL_1_R expression (Northern blot, in situ hybridization)	[32]
Insulinoma Rat RINm5F cell line	- GAL moderately suppressed insulin accumulation, but did not affect cell proliferation	[138]
	- Pancreatic beta-cells: GAL inhibited adenylate cyclase activity and insulin secretion	[53]
Insulinoma Mouse	- Beta TC-1 cells: GAL, released from sympathetic nerve terminals, inhibited pro-insulin gene expression stimulated by glucagon-like peptide-I (Northern blot)	[139]
Neuroblastic tumors Human	- GAL mRNA, GAL immunoreactivity and GAL binding sites expression (IH, in situ hybridization)	[137]
	- Low level of GAL binding sites correlated with survival; GAL/GALR expression related to tumor differentiation stage (RIA, IH, in situ hybridization)	[136,137]
Neuroblastoma Human	- No correlation between prognosis/tumor markers and GAL concentration	[136]
	- GAL expression; GAL2R mRNA was less common than GAL1R mRNA (IH, in situ hybridization)	[104]
	- GAL1R/GAL3R highly expressed; GAL promoted tumor growth (IH, in situ hybridization)	[137]
Neuroblastoma Human IMR32 cell line	- Dense core secretory vesicles: coexistence of GAL and beta-amyloid (IH)	[140]
Neuroblastoma Human SH-SY5Y cell line	- GAL2R mediated apoptosis. GAL antiproliferative potency: 100-fold higher in SY5Y/GAL2R cells than in SY5Y/GAL1R cells	[12]
	- GAL2R transfection: cell proliferation was blocked and caspase-dependent apoptotic mechanisms induced	[12]
Neuroblastoma Rat B104 cell line	- GAL, GAL2R and GAL3R mRNAs were detected, but not GAL1R mRNA (reverse transcription-PCR)	[141]
	- GAL promoted cell proliferation	
Paraganglioma Human	- GAL expression (IH)	[108,112,142]
Paraganglioma Human carotid body	- GAL was detected in 18% of tumors (IH)	[108]
Paraganglioma Human jugulo tympanic	- GAL was detected in 40% of tumors (IH)	[108]
Phaeochromocytoma Human	- High GAL2R mRNA expression (Western blot)	[143]
	- Higher GAL concentration than in normal adrenal glands (RIA)	[144]
Phaeochromocytoma Rat PC12 cell line	- GAL inhibited cell proliferation and GAL_1_R, GAL_2_R and GAL_3_R mRNA expression, but not GAL mRNA (reverse transcription-PCR)	[141]
Pituitary adenoma Human	- GAL/GALR expression correlated with tumor stage (IH)	[101]
Pituitary adenoma Human	- High GAL_3_R levels found in some patients who relapsed shortly after surgical intervention (q-PCR)	[145]
Pituitary adenoma Rat	- GAL promoted pituitary cell proliferation and tumor development	[38]
Pituitary adenoma Rat MtTW-10 cell line	- Estradiol increased GAL mRNA level	[146]
Prolactinoma Rat	- GAL concentration increased and GAL promoted tumor development	[147,148]
	- Levonorgestrel decreased GAL mRNA expression and GAL-expressing cells (IH, in situ hybridization)	[149]
Small-cell lung cancer Human H345, H510 cell lines	- GAL, via GAL_2_R, mediated cell proliferation	[88,150]
Small-cell lung cancer Human H69, H510 cell lines	- GAL, via GAL2R, activated G proteins and promoted cell proliferation	[88]
	- GAL increased the levels of inositol phosphate and intracellular Ca^2+^ and promoted cell growth	[151]
Small-cell lung cancer Human H345, H510 cell lines	- Ca^2+^-mobilizing peptides (e.g., GAL) promoted cell growth. Broad spectrum antagonists directed against multiple Ca^2+^-mobilizing receptors inhibited cell growth	[150,152]
Small-cell lung cancer Human H69, H345, H510 cell lines	- GAL, via the p42MAPK pathway, promoted cell growth. Protein kinase C inhibitors blocked cell growth induced by GAL	[153,154]
Small-cell lung cancer Human SBC-3A cell line, mouse SBC-3A tumor	- SBC-3A cells secreted the pre-pro-GAL precursor which was extracellular processed to GAL1-20 by plasmin	[155,156]
Somatotroph adenoma Human	- Low GAL level (RIA)	[102]
	- GAL increased circulating growth hormone level and growth hormone-producing tumors expressed GAL (IH)	[157]
	- GAL blocked growth hormone release	[158]
Somatotroph adenoma Rat GH1 cell line	- GAL inhibited growth hormone release	[159]
Somatotroph adenoma Mouse	- GAL mRNA level and peptide concentration increased	[147]
	- GAL secretion increased	[160]
Thyrotroph adenoma Rat	- GAL gene expression blocked	[147]
Thyrotroph adenoma Mouse	- GAL synthesis inhibited	[160]

IH: immunohistochemistry; q-PCR: quantitative real time PCR; RIA: radioimmunoassay.

**Table 2:** Involvement of the galaninergic system in gastric and colorectal cancer.

	Actions/Presence	References
Gastric Cancer		
Human	- Fibers containing GAL: increased in longitudinal muscle layer, lamina muscularis mucosae and neoplastic proliferation vicinity (IH)	[175]
	- Myenteric plexus: neurons showed a high expression of caspases 3/8 and low GAL expression (IH)	[175]
	- GAL/GAL1R level reduced	[176]
	- GAL2R/GAL3R level unchanged (RT-PCR)	[176]
	- Lower level of GAL in pre-operative samples (and plasma) when compared with that found in post-operative samples or in healthy donors. Gastric cancer tissues: GAL/GAL1R level was lower compared with that found in adjacent regions GAL2R/GAL3R: no change (Western blot; RT-PCR; ELISA)	[176]
	- GAL low level: used as biomarker. GAL protein/mRNA level related to tumor size, tumor node metastasis stage and lymph node metastasis	[176]
Human Gastric cancer cell lines	- GAL expression decreased: restored with a demethylating agent. GAL hypermethylation: impaired GAL tumor suppressor action. GAL downregulation: due to epigenetic inactivation (Q-MSP, Western blot)	[177]
	- GAL: decreased cell proliferation	[178]
Rats	- GAL blocked gastric carcinogenesis by inhibiting antral epithelial cell proliferation	[13]
Colorectal Cancer (CRC)		
Human	- GAL/GAL1R silencing: apoptosis in drug-sensitive/resistant cell lines and enhanced the effects mediated by chemotherapy. GAL mRNA: overexpressed. High GAL level: related to poor disease-free survival of early-stage CRC patients (IH, ELISA, RT-PCR, Western blot)	[7,106,117,121]
	- Enteric nervous system: number of neurons containing GAL increased in regions located close to the tumor (IH) (IH, RT-PCR, ELISA)	[8]
	- CRC patients: more GAL-immunoreactive neurons in comparison to healthy samples (IH, ELISA)	[121]
	- GAL in the vicinity of cancer cell invasion (IH, ELISA)	[121]
	- Blood samples: increased GAL concentration. High GAL level: cancer cells. Lowest GAL level: muscular layer placed distant from tumors. GAL: CRC tumor biomarker (ELISA, IH)	[179]
	- GAL mRNA level: related to adenocarcinoma size/stage. Correlation between higher GAL expression and shorter disease-free survival (RT-PCR)	[106,117]
	- CRC cells showed a high GAL expression: more malignant and involved in tumor recurrence. High GAL expression: spread of cancer stem cells (metastasis) (RT-PCR)	[180]
	- High GAL expression: associated with poor prognosis (stage II) and tumor recurrence. GAL expression: related to CRC aggressive behavior (RT-PCR)	[180]
Human (tissue and cell lines)	- CRC cells/tissues: higher GAL levels than non-tumor cells/tissues	[106,117,179,180]
	- CRC tissue: increased GAL gene/protein expression. CRC cell lines: GAL/GAL1R silencing promoted apoptosis. GAL1R silencing promoted FLIPL down-regulation (IH, ELISA, RT-PCR)	[106,117,121]
Human HCT116 cell line	- Cells overexpressing GAL_2_R were more chemosensitive to bevacizumab than control cells	[181]
Rat	- GAL decreased the incidence of colon tumors	[182]

IH: immunohistochemistry; Q-MSP: quantitative methylation-specific PCR; RT-PCR: real time-PCR.

**Table 3:** Involvement of the galaninergic system in head and neck squamous cell carcinoma.

	Actions/Presence	References
Human	- High GAL level (RT-PCR)	[120]
	- GAL1R gene promoter: frequently methylated (Q-MSP)	[191]
	- Methylation status of some peptide-encoding genes, including GAL, is related with survival and recurrence. Methylation changes: possible molecular marker for HNSCC risk/prognosis (Q-MSP)	[192]
	- GAL/GALR epigenetic variants: markers for prognosis prediction (Q-MSP)	[193,194]
	- Poor survival: associated with methylation of GAL/GAL1R genes. Hypermethylation: inactivation of GAL/GAL1R/GAL2R genes (Q-MSP)	[195]
Human Cell lines	- Apoptosis: mediated by GAL2R but not by GAL1R. GAL1R/GAL2R: tumor suppressors in a p53-independent manner	[11]
	- GAL2R transfection into HNSCC cells: cell proliferation inhibited. GAL2R re-expression: blocked cell proliferation (showing mutant p53)	[113,196,197]
	- GAL1R/GAL2R negative HNSCC cells: GAL1R re-expression suppressed tumor cell proliferation via ERK1/2-mediated actions on cyclin-dependent kinase inhibitors and cyclin D1	[113,197]
	- GAL/GAL1R blocked HNSCC and oral tumor cell proliferation by cell-cycle arrest (RT-PCR, ELISA, Q-MSP)	[123,177,196,198]
	- GAL1R blocked tumor cell proliferation through the activation of ERK1/2	[196]
	- GAL2R promoted an antitumor effect by inducing cell cycle arrest and apoptotic mechanisms (caspase 3-dependent)	[197]
	- GAL2R suppressed HNSCC cell viability. HEp-2 cells: GAL2R mediated apoptotic mechanisms (caspase-independent) by downregulating ERK1/2 and inducing Bim	[199]
Human Cell lines, tumor samples	- GAL2R overexpression: favored survival/proliferation by activating PI3K/Akt and MAPK/ERK-dependent pathways. Ras-related protein 1 (Rap1): involved in HNSCC progression.	[122]
	- GAL/GAL1R: tumor suppressor. GAL1R absent in some cell lines (Q-MSP, RT-PCR)	[177,178,198]
	- GAL1R promoter: widely hypermethylated and related to reduced GAL1R expression. GAL1R/GAL2R hypermethylation: associated with higher recurrence rate and reduced disease-free survival (RT-PCR, Q-MSP)	[191,194,198,200]
	- GAL1R methylation status: potential biomarker for predicting clinical outcomes. Methylation: related to carcinogenesis and decreased GAL1R expression (RT-PCR, Q-MSP)	[193,194,198]
Human (cell lines) Mouse	- GAL (released from nerves) activated GAL_2_R expressed in tumor cells inducing NFATC2-mediated transcription of cyclooxygenase-2 and GAL. GAL released from tumor cells promoted neuritogenesis, favoring perineural invasion	[84]
Mouse	- GAL_2_R promoted tumor angiogenesis through the p38-MAPK-mediated inhibition of tristetraprolin (TTP), leading to an enhanced secretion of cytokines. GAL_2_R activated Ras-related protein 1b (Rap1B) favoring a p38-mediated inactivation of TTP, which acted as a destabilize cytokine transcript	[201]

Q-MSP: quantitative methylation-specific PCR. RT-PCR: real-time PCR.

**Table 4:** Involvement of the galaninergic system in glioma.

	Actions/Presence	References
Human	- GAL/GAL3R expression: no correlation with oligodendroglial, astrocytic and mixed neural–glial tumors	[30]
	- High-grade glioma (WHO grade IV): related to GAL3R expression	[30]
	- Endothelial/immune cells: GAL3R expression. Around blood vessels: GAL1R/GAL2R not observed (IH)	[30]
	- GAL1R, followed by GAL3R; GAL2R absent (astrocytic/oligodendroglia tumors) (IH, autoradiography, reverse transcription-PCR)	[30,118]
	- Glioma-associated macrophages: GAL3R expression (quantitative PCR)	[59,204]
	- No correlation between proliferative activity and GAL/GAL binding levels (IH, autoradiography, reverse transcription-PCR)	[118]
	- Cerebrospinal fluid (glioblastoma): reduced GAL level	[203]
Human Mice	- GAL blocked, via GAL_1_R, the proliferation of glioma cells and tumor growth. These effects were mediated through ERK1/2 signal activation. No cytotoxic/apoptotic effect was observed	[205]

IH: immunohistochemistry.

**Table 5:** Involvement of the galaninergic system in other cancers.

	Actions/Presence	References
Breast cancer Human	- GAL/pre-pro-GAL mRNA level expression. GALN gene: unlike candidate oncogene (Northern blot)	[101,206]
Carcinoma (cardiac, esophageal) Human	- Fibers containing GAL contacted closely with cancer cells (IH)	[207]
Endometrial cancer Human	- GAL_1_R DNA methylation indicated malignancy (q-PCR)	[208]
Bladder cancer Human	- GAL_1_R gene methylation involved in prognosis	[209]
Salivary duct carcinoma Human	- GAL_1_R/GAL_2_R: therapeutic targets/prognostic factors. GAL_1_R/GAL_2_R methylation rates correlated with overall survival decrease (IH, Q-MSP)	[210]
Melanoma Human	- GAL/GAL_1_R expression (IH)	[101,119]
Pancreas Human	- GAL promoted SW1990 cell proliferation	[211]
Pancreas Rat	- GAL blocked carcinogenesis and decreased norepinephrine level (IH, HPLC)	[212]

HPLC: high-performance liquid chromatography; IH: immunohistochemistry; Q-MSP: quantitative methylation-specific PCR; q-PCR: quantitative real-time PCR.

**Table 6:** Proliferative and antiproliferative actions of the galaninergic system in different tumors. +: action mediated by GAL, GAL_1_R, GAL_2_R or GAL_3_R.

Cancer	GAL	GAL1R	GAL2R	GAL3R	References
**A. Proliferative action**					
Colorectal	+	+			[8,30,106,117,185]
Glioma				+	[30]
Head and neck squamous cell carcinoma	+		+	+	[84,122,123,201]
Neuroblastoma	+				[137,141]
Pancreas	+				[211]
Pituitary adenoma	+			+	[38,145]
Prolactinoma	+				[148,160]
Small-cell lung cancer	+		+		[88,113,150,151,173]
**B. Antiproliferative action**					
Colorectal	+				[182]
Endometrial		+			[208]
Gastric	+				[13,177,178]
Gastrointestinal	+				[101]
Head and neck squamous cell carcinoma	+	+	+		[13,16,120,170,184,190,192]
Neuroblastoma			+		[12]
Pancreas	+				[212]
Phaeochromocytoma	+				[141]
Salivary duct carcinoma		+	+		[210]
